# Correlates of type 2 diabetes and glycaemic control in adults in Saudi Arabia a secondary data analysis of the Saudi health interview survey

**DOI:** 10.1186/s12889-020-08597-6

**Published:** 2020-04-17

**Authors:** Thamer Al Slamah, Barbara I. Nicholl, Fatima Y. Alslail, Leanne Harris, Deborah Kinnear, Craig A. Melville

**Affiliations:** 1 Human Health Department, College of Applied Medical Sciences, Qassim University, Buraydah, Kingdom of Saudi Arabia; 2 General Practice and Primary Care, Institute of Health and Wellbeing, University of Glasgow, 1 Horselethill Road R202 House 2, Glasgow, G12 9LX UK; 3 College of Medicine, Veterinary and Life Science, University of Glasgow, Glasgow, UK; 4 Director of the National Diabetes Control and Prevention Program, Ministry of Health, Riyadh, Kingdom of Saudi Arabia; 5 School of Medicine, Dentistry & Nursing, University of Glasgow, Glasgow, UK; 6 Mental Health and Wellbeing, Institute of Health and Wellbeing, Glasgow, UK

**Keywords:** Diabetes, Type 2 diabetes, Self-care, Self-management

## Abstract

**Background:**

There is evidence that type 2 diabetes self-management programmes may have a positive impact on health outcomes of adults living in Gulf countries. However, none of the programmes evaluated were developed using evidence about the specific needs of adults with Type 2 diabetes living in the Gulf countries. This study is part of a wider programme of research, which uses a cultural adaptation framework to generate information on how to tailor type 2 diabetes self-management to the Saudi context.

**Methods:**

Secondary data analysis of the Saudi Health Interview Survey (SHIS) (*N* = 10,821) was conducted. Bivariate and multivariate logistic regression modelling assessed factors associated with type 2 diabetes and its control / self-management including sociodemographic factors (e.g. age, gender), lifestyle (e.g. diet, physical activity), and health seeking behaviours (e.g. chronic illnesses, health services).

**Results:**

7% (*N* = 808) of all participants had type 2 diabetes (59% male), however it represents 35% at or above 55 years. In multivariate analysis at older age, being overweight or obese, male, having hypertension, and reporting a reduction in health status in the 12 months prior to questionnaire completion, were significantly associated with having type 2 diabetes. Participants who reported walking for more than 10 min per day were less likely to report type 2 diabetes. Unexpectedly there was a significant association between type 2 diabetes and lower frequency of fast food intake, while increased fruit and vegetable intake was associated with poor glycaemic control.

**Conclusions:**

Being overweight and/or hypertensive are concomitant with type 2 diabetes in Saudi Arabia. Any self-management programmes for type 2 diabetes patients with either of these conditions should be tailored accordingly. Walking behaviours should be prioritised in Saudi self-management programmes. Prediabetes management programmes may be of special importance to the Saudi community.

## Background

The prevalence of type 2 diabetes in Saudi Arabia has been increasing due to socioeconomic changes that have affected lifestyle habits including changes in diet and physical activity [1]. Approximately 13% of Saudis [2] are thought to have type 2 diabetes compared to the 2. 8-4.4% global prevalence [3], while one in 10 of the remaining Saudi population is thought to be at risk of developing diabetes (prediabetes) [2]. This high prevalence of type 2 diabetes is also associated with a high prevalence of cardiovascular disease (CVD) and premature mortality [4]. 42% of mortalities in the Saudi population are associated with CVD. The average individual healthcare expenditure of a diabetes patient is 10-fold that of the average Saudi individual who does not have type 2 diabetes [5].

Globally, diabetes self-management programmes have led to a significant reduction of the economic burden associated with type 2 diabetes [6], and have improved the health and quality of life of diabetes patients [6]. We previously carried out a systematic review on type 2 diabetes self-management training programmes in Gulf countries [7], including Saudi Arabia. The review found that self-management programmes have the potential to improve the health and wellbeing of individuals with type 2 diabetes [7]. Five out of the eight studies included were from Saudi Arabia and used different approaches ranging from education, regular attendance at specialised clinics for check-ups, dietary advice, physical exercise or a combination of these. Half (four) of these studies reported a 0. 5-2% drop in HbA1c following self-management programmes. However, these studies did not assess key desired outcome elements of self-management programmes such as the ability to transmit and acquire skills. They also lacked a proper pilot study for any of the available structured self-management education programmes elsewhere. For example, in the UK, the Diabetes Education Self-management for Ongoing and Newly Diagnosed (DESMOND) programmes proving a success [8] due to its focus on equipping trainers and educators to provide high quality self-management programmes in a manner suited to their audience [9, 10]. DESMOND recently introduced the ‘Let’s prevent diabetes’ program, which encourages self-management programmes for those who are at risk of developing diabetes with an aim of preventing or at least delaying the progression of the condition [11]. Other self-management programmes such as Diabetes Self-Management Education (DSME) in the USA, have addressed cultural adaptation of the programme within different ethnicities at the same geographical locations and managed to increase compliance through including families as a whole in the programme, liaising with religious leaders and providing familiar analogues to the positive and negative effects of some behaviours or habits [12]. Therefore, for any of these programmes to be more successful in a new community such as within Saudi Arabia, there is a need for specific cultural adaptation of programme content [13, 14]. DESMOND has the potential to be transferred to other countries such as Saudi Arabia; however, careful cultural adaptation of the programmes necessary to shape the structure of the programme with a clearer focus on specific self-management skills that can impact on health improvement for local individuals [15].

Cultural adaptation of self-management programmes and adjusting them to the available healthcare resources, healthcare needs, sociodemographic characteristics (e.g. age, gender, education, occupation conditions) of populations and the capacities of both potential educators and patients are crucial to the success of these programmes [14]. Identifying the unmet health needs of the population would allow for more appropriate targeting of healthcare resources [16–18] and the development of a targeted self-management programme. Therefore, the overall aim of our research is to develop a culturally relevant DSME/DESMOND model for Saudi Arabia. The theoretical framework provided by Kumpfer’s cultural adaptation model, which gives a progressive sequence of nine stages was used here for the research programme [19]. Stage one involved a systematic review of the available literature on type 2 diabetes self-management training programmes in Gulf countries as discussed above [7]. The current study is the second stage in our overall aim to develop a culturally relevant DSME model for Saudi Arabia by determining the needs of the population. Our aim is to inform the development of a culturally relevant type 2 diabetes self-management programme using population level data including sociodemographic factors, lifestyle (e.g. diet, physical activity), and health seeking behaviours (e.g. chronic illnesses, health services. The aims of this study were to identify:

1. How sociodemographic characteristics, lifestyle, and health-seeking behaviours vary between those with type 2 diabetes compared to the rest of the population sample. And which, if any, of the factors outlined are associated with type 2 diabetes.
2. How sociodemographic characteristics, lifestyle, and health-seeking behaviours vary between those with well-controlled and those with poorly-controlled type 2 diabetes. And which, if any, of the factors outlined are associated with poorly controlled diabetes.

## Methods

### SHIS study design

The Saudi Health Interview Survey (SHIS) [20], conducted in 2013, covering all 13 regions in the Kingdom of Saudi Arabia (Al Riyadh; AlQassim; Makkah Al Moukarrama; Tabuk; Hail; Al-Jouf; Al-Baha; Eastern Region; Northern Borders; Madinah; Jezan, and; Aseer; Najran) on adults aged 15 years or older. A multistage stratified probability sample was developed to recruit the study participants. Stratification was based on the 13 regions of the Kingdom. A total of 12,000 households were randomly selected and contacted from the 13 administrative regions. A total of 10,827 participants completed the survey and were invited to local health clinics. All survey weights were post-stratified to the general Saudi population and to the composition of the selected adults. Physical measures were taken including height (cm), weight (kg), waist circumference (cm), blood pressure (mmHg), heart rate (pulses/min) and respiratory rate (breathes/min). A questionnaire and medical record review were performed for each participant. The questionnaire provided a self-report of sociodemographic characteristics, lifestyle including nutrition, habits such as tobacco use, physical activity, and health-seeking behaviours (e.g. routine regular checks versus admissions or emergency visits). Medical records and questionnaires were used to record chronic diseases including type 2 diabetes. Participants were referred to local clinics in hospitals and primary care health centres for blood samples to be investigated for lipid profile, Vitamin D and HbA1c.

### Secondary data analysis

Variables collected for SHIS, of clinical relevance to type 2 diabetes and diabetes control, were carefully selected for secondary analysis by a consensus process involving all members of the research team. These variables included sociodemographic characteristics, lifestyle, health condition and chronic illness, and health-seeking behaviours relevant to the research question and are detailed in Table 1 and Additional file 1.

**Table 1:** Sociodemographic, lifestyle and health-seeking characteristics of participants with and without type 2 diabetes

	Type 2 diabetes ***N*** = 808		No type 2 diabetes ***N*** = 10,013		Odds Ratio (95% CI)	***p***-value
	Total n	n (%)	Total n	n (%)		
***Sociodemographic characteristics***						
**Gender**	808		10,013			
Male		477 (59)		4819 (48.1)	REF	
Female		331 (41)		5194 (51.9)	0.64 (0.55, 0.74)	< 0.001
Missing	0		0			
**Age**	802		9933			
15-54		352 (43.9)		8646 (87)	REF	
≥ 55		450 (56.1)		1287 (13)	8.58 (7.38, 9.99)	< 0.001
Missing N%?	6 (%)		80 (%)			
**Marital status**	806		9978			
Married		619 (76.8)		4613 (64.3)	REF	
Not married		187 (23.2)		3565 (35.7)	0.54 (0.45, 0.64)	< 0.001
Missing	2		35			
**Education level**	806		9991			
Primary school or less		477 (59.2)		2847 (28.5)	REF	
Elementary or high school College degree or higher education completed		329 (40.8)		7177 (71.5)	0.27 (0.23, 0.31)	< 0.001
Missing	2		22			
**BMI**	797		9915			
Overweight or obesity		687 (86.2)		6466 (65.2)	REF	
Normal weight		110 (13.8)		3449 (34.8)	0.30 (0.24, 0.36)	< 0.001
Missing	11		98			
***Lifestyle characteristics***						
**Smoking status**	808		10,013			
Previous smoker		59 (7.3)		404 (4)	REF	
Current smoker		114 (14.1)		1252 (12.5)	0.62 (0.44, 0.87)	< 0.001
Never smoked		635 (78.6)		8357 (83.5)	0.52 (0.39, 0.69)	< 0.001
Missing	0		0			
**Dietary fat intake**	793		9828			
Vegetable or olive oils		731 (92.2)		8993 (91.5)	REF	
Animal fat or margarine or none in particular		62 (7.8)		835 (8.5)	0.91 (0.69, 1.19)	0.509
Missing	15		185			
**Dietary meat intake**	744		9048			
0–7		559 (75.1)		5859 (64.8)	REF	
8+		185 (24.9)		3189 (35.2)	0.60 (0.51, 0.72)	< 0.001
Missing	64		965			
**Dietary fruit and vegetable intake**	726		8885			
0–2		377 (51.9)		4628 (52.1)	REF	
3+		349 (48.1)		4257 (47.9)	1.00 (0.86, 1.17)	0.938
Missing	82		1128			
**Dietary fast food intake**	698		8661			
0–1		603 (86.4)		5557 (64.2)	REF	
2+		95 (13.6)		3104 (35.8)	0.28 (0.22, 0.35)	< 0.001
Missing	110		1352			
**Work physical activity**	808		10,013			
No		800 (99)		9791 (97.8)	REF	
Yes		8 (1)		222 (2.2)	0.44 (0.21, 0.89)	0.021
Missing	0		0			
**Sport physical activity**	808		10,013			
No		771 (95.4)		8878 (88.7)	REF	
Yes		37 (4.6)		1135 (11.3)	0.37 (0.26, 0.52)	< 0.01
Missing	0		0			
**Walking behaviour**	802		9904			
No		394 (49.1)		3991 (40.3)	REF	
Yes		408 (50.9)		5913 (59.7)	0.69 (0.60, 0.80)	< 0.01
Missing	4		109			
**TV viewing time**	709		8644			
0–3 Hours		459 (64.7)		5090 (58.9)	REF	
4+ Hours		250 (35.3)		3554 (41.1)	0.78 (0.66, 0.91)	0.002
Missing	9		1369			
**Sitting time**	732		8674			
0–4 Hours		345 (47.7)		4984 (57.5)	REF	
5+ Hours		387 (52.3)		3690 (42.5)	1.48 (1.27, 1.72)	< 0.001
Missing	76		1339			
***Health seeking behaviours***						
**Hypertension**	808		9945			
No		34 (4.2)		2610 (26.2)	REF	
Yes		774 (95.8)		7335 (73.8)	8.10 (5.72, 11.45)	< 0.001
Missing	0		68			
**Chronic disease diagnosis**	755		9673			
No		560 (74.2)		8923 (92.2)	REF	
Yes		195 (25.8)		750 (7.8)	4.14 (3.46, 4.95)	< 0.001
Missing	53		340			
**Self-reported health status**	806		9980			
Very good or good		627 (77.8)		9278 (93)	REF	
Fair or poor		179 (22.2)		702 (7)	3.77 (3.14, 4.53)	< 0.001
Missing	2		33			
**Self-reported health status compared with 12 months**	794		9896			
Better or same		563 (70.9)		8482 (85.7)	REF	
Worse		231 (29.1)		1414 (14.3)	2.46 (2.09, 2.89)	< 0.001
Missing	14		117			
**Visited health service**	519		6781			
Illness or injury		66 (12.7)		1303 (19.2)	REF	
Other services		453 (87.3)		5478 (80.8)	1.63 (1.25, 2.12)	< 0.001
Missing	289		3232			

*REF* Reference category for statistical analysis, *CI* Confidence interval, *BMI* Body mass index

Variables including “visiting physician or health professional to manage diabetes”, “self-assessed blood sugar at home”, “distance to nearest health facility,” and “time needed to reach nearest health facility”, were excluded due to high frequency of missing data, rendering them unusable for analysis (> 75% of missed data). While all variables included had a maximum of 10% missing data. The data was cleaned through visually inspecting histograms for spurious data points, and outliers. Categorical responses, were classified to binary or two responses only (Additional file 1), apart from smoking where a third response (never smoked) was considered clinically important [21]. Continuous data, such as age, frequency of fruit or meat servings, were split according to the median value [22], others such as HbA1c, physical activity through leisure time sports activity and occupation activity were divided according to the following definitions: HbA1c equal to or more than seven (poor control) or less (good control) [23, 24] and 150 min or more / week for good physical activity of sport or work [25]. One of the known disadvantages of categorising the data in this manner is the potential loss of significance power of some factors [26]. However, where possible categorisation of variables was based on accepted clinical benchmarks as outlined above.

### Data analysis

SPSS 24 IBM statistical package (SPSS IBM, New York, NY, USA) was used to conduct all analysis.

Descriptive statistics were used to compare the frequency distribution of sociodemographic characteristics, lifestyle, and health-seeking behaviours between participants with and without type 2 diabetes.

Separate analyses compared participants with well controlled type 2 diabetes, to poorly controlled groups using HbA1c. As the above definition, those with HbA1c < 7 were considered as well controlled and those with HbA1c ≥7 as poorly controlled [23, 24].

For both research questions, differences between the two groups were examined for statistical significance using binary logistic regressions in a six-step model-building approach using a series of bivariate and multivariate analyses [27].

#### Step 1

A series of bivariate analyses between each predictor variable and the outcome variable for each model were conducted. This purposeful selection of variables was to identify variables to be taken forward to the multivariate analysis. A test significance of *p*-value of < 0.25 was used for this initial stage to screen variables for their potential relevance to type 2 diabetes or glycaemic control (dependent variables). Only variables that met the criteria were taken forward to the multivariate analysis [27].

#### Step 2

A multivariate binary regression model (larger model) was fit to the variables, that met the criteria in stage one (*p* <  0.25). A backward stepwise least squares logistic regression model was conducted to sequentially remove variables that were non-significant, developing a smaller model which contained only statistically significant variables (Wald statistic *p* <  0.05). The fit of this smaller model was compared to the larger multivariate regression model (calculated by the difference in log-likelihoods and interpreted using the chi-squared distribution) [28].

#### Step 3

The coefficient values (beta) for each variable in the smaller model were compared to the beta values in the larger model. If a change in beta of ±20% between the two models was observed, this indicated that variables excluded were important to the model, in terms of adjusting an effect. These were then entered back into the smaller multivariate model.

#### Step 4

Variables that were excluded at stage one were entered (forced entry) one at a time into the smaller multivariate model (identified at the end of step three) to test their contribution to the model (assessed using the Wald statistic *p* <  0.05). Although they were not independent predictors of type 2 diabetes or glycaemic control at stage one, re-entering these variables into the smaller model tested whether they make a significant contribution to the model in the presence of other contributing variables.

#### Step 5

The model at the end of step four is the preliminary main effects model. Interactions between the variables in the preliminary main effects model were assessed for significance, one at a time using log-likelihoods to test their significance (*p* <  0.05). Interactions that were conceptually plausible and statistically significant were entered (forced entry) into the smaller model. The significance of all included interactions was then assessed using the Wald statistic, with any non-significant interactions (*p* > 0.05) removed from the model. The variables remaining in the model represented the final main effects model.

#### Step 6

The overall fit of the final main effects model was assessed by the Hosmer-Lemeshow goodness of fit statistic [29]. A large *p*-value (*p* > 0.10) indicate a good fit of the model relevant to the data [29]. The final model was assessed to ensure it met the assumptions of logistic regression. Residuals were checked using standardised residuals (< 5% outside ±1.96) and Cook’s assumption (< 1) [28]. The assumption of multicollinearity (tolerance < 0.10 and VIF > 10) was also assessed [28].

## Results

### Participant characteristics

#### Type 2 diabetes

Of the 10,821 participants completing the SHIS survey, 808 participants (7.5%) were identified as having type 2 diabetes [41% female (*n* = 331), mean age = 38.38 ± 16.1 years]. Participants with type 2 diabetes were more likely to be overweight or have obesity (86.2% of type 2 diabetes sample had a BMI ≥25 kg/m^2^ compared to 65.2% of participants who did not have type 2 diabetes). Based on self-report, participants with type 2 diabetes were also more likely to have hypertension (95.8%) compared to patients without diabetes (67.5%). All characteristics are summarised in Table 1.

#### Predictors of type 2 diabetes

##### Bivariate analysis **(step 1)**

The bivariate analysis (Table 1) illustrates that older married males, who are overweight, consume higher meat or fast food, while less active at work or practice less sport, viewing TV or setting longer periods and suffer from hypertension, chronic disease or reported themselves to have poor health or felt worse health comparing with 12 months earlier and / or not frequently visiting health services were more likely to have type 2 diabetes.

### Multivariate analysis and final model (steps 2-6)

Additional file 2 provides beta percentages between the smallest and largest interactions. All interaction results are provided in Additional file 3. The final multivariate model (Table 2) found that participants of older age (≥55 years), with hypertension, chronic disease, and/or poorer self-reported health status compared with 12 months ago were significantly more likely to have type 2 diabetes. Females, and individuals with normal weight, those who ate more fast food (≥2 times per week) and walked more than 10 min per day, were less likely to have type 2 diabetes.

**Table 2:** Final multivariate logistic regression model for the association between sociodemographic, lifestyle and health-seeking behaviours and type 2 diabetes

Variables	Β	SE	Odds Ratio (95% CI)	***p***-value
**Gender**				
Male	REF	REF	REF	
Female	−0.64	0.09	0.52 (0.43, 0.63)	< 0.001
**Age**				
15-54	REF	REF	REF	
≥ 55	1.62	0.09	5.09 (4.19, 6.18)	< 0.001
**BMI**				
Overweight or obesity	REF	REF	REF	
Normal weight	− 0.99	0.12	0.37 (0.29, 0.47)	< 0.001
**Hypertension**				
No	REF	REF	REF	
Yes	1.52	0.20	4.58 (3.07, 6.82)	< 0.001
**Chronic disease diagnosis**				
No	REF	REF	REF	
Yes	0.50	0.11	1.65 (1.32, 2.07)	< 0.001
**Self-reported health status compared with 12 months** **Status**				
Better or same	REF	REF	REF	
Worse	0.47	0.10	1.61(1.31, 1.97)	< 0.001
**Dietary fast food intake**				
0–1 per week	REF	REF	REF	
2+ per week	−0.69	0.12	0.49 (0.39, 0.63)	< 0.001
**Walking behaviour more than 10 mints per day**				
No	REF	REF	REF	
Yes	−0.32	0.09	0.72 (0.60, 0.86)	< 0.001
**Interaction**				
**Age (**15-54)* Chronic disease diagnosis (No)	REF	REF	REF	
Age(≥ 55) * Chronic disease diagnosis (Yes)	−0.89	0.22	0.40 (0.26, 0.63)	< 0.001
**Age(**15-54)* **Self-reported health status compared with 12 months (Better or same)**	REF	REF	REF	
**Age (≥ 55)** * **Self-reported health status compared with 12 months (Worse)**	−0.62	0.20	0.53 (0.36, 0.79)	0.002

*REF* Reference category, *SE* Standard error, *CI* Confidence interval, *BMI* Body mass index. B, beta coefficient

Significant interactions were established between age (≥55 years) and participants with chronic disease and/or self-reported worse health status after 12 months. Hosmer and Lemeshow test for goodness of fit for the final model was 0.450, indicating good fit (*p* > 0.10). Collinearity diagnostic and the Tolerance test also confirmed a goof fit of the model.

### Diabetes control

Only 391 individuals with type 2 diabetes (48.4%) had a measured HbA1c. There were no statistically significant differences in the sociodemographic factors, lifestyle, and health seeking behaviours between the 164 participants (41%) defined as having poor glycaemic control and the 227 participants with good glycaemic control. The majority of this sample (62%) was only educated up to primary level or less. However, the percentage of those who were educated to elementary up to higher education was higher within the good control group (41% versus ~ 35%). However, 57.6% of the poor control group ate more than three portions of fruits and vegetables, which was higher than 45.1% in the other group. All characteristics are provided in Table 3.

**Table 3:** Sociodemographic, lifestyle and health-seeking characteristics of participants with poor and good glycaemic control

	Poor glycaemic control ***N*** = 164		Good glycaemic control ***N*** = 227		Odds Ratio (95% CI)	***p***-value
	Total n	n (%)	Total n	n (%)		
***Sociodemographic characteristics***						
**Gender**	164		227			
Male		98 (59.8)		133 (58.6)	REF	
Female		66 (40.2)		94 (41.4)	0.95 (0.63, 1.43)	0.817
Missing	0		0			
**Age**	164		225			
15-54		73 (44.5)		94 (41.8)	REF	
≥ 55		91 (55.5)		131 (58.2)	0.89 (0.59, 1.34)	0.591
Missing	0		2(%)			
**Marital status**	164		227			
Married		130 (79.3)		168 (74)	REF	
Not married		34 (20.7)		59 (26)	0.74 (0.46, 1.20)	0.228
Missing	0		0			
**Education level**	164		227			
Primary school or less		107 (65.2)		134 (59)	REF	
Elementary or high school College degree or higher education completed		57 (34.8)		93 (41)	0.76 (0.50, 1.16)	0.212
Missing	0		0			
**BMI**	162		226			
Overweight or obesity		144 (88.9)		196 (86.7)	REF	
Normal weight		18 (11.1)		30 (13.3)	0.81 (0.43, 1.52)	0.523
Missing	2		1			
***Lifestyle characteristics***						
**Smoking status**	164		227			
Previous smoker		17 (10.4)		18 (7.9)	REF	
Current smoker		17 (10.4)		26 (11.5)	1.44 (0.58, 3.55)	0.424
Never smoke		130 (79.3)		183 (80.6)	1.32 (0.66, 2.67)	0.425
Missing	0		0			
**Dietary fat intake**	161		224			
Vegetable or olive oils		138 (85.7)		204 (91.1)	REF	
Animal fat or margarine or none in particular		23 (14.3)		20 (8.9)	1.55 (0.81, 2.97)	0.182
Missing	3		3			
**Dietary meat intake/week????**	156		206			
0–7		114 (73.1)		156 (75.7)	REF	
8+		42 (26.9)		50 (24.3)	1.14 (0.71, 1.85)	0.566
Missing	8		21			
**Dietary fruits and vegetables intake/week??**	151		206			
0–2		64 (42.4)		113 (54.9)	REF	
3+		87 (57.6)		93 (45.1)	1.65 (1.08, 2.52)	0.020
Missing	13		21			
**Dietary fast food intake/week???**	146		198			
0–1		122 (83.6)		181 (91.4)	REF	
2+		24 (16.4)		17 (8.6)	2.09 (1.08, 4.06)	0.026
Missing	18		29			
**Work physical activity**	164		227			
No		161 (98.2)		226 (99.6)	REF	
Yes		3 (1.8)		1 (0.4)	4.21 (0.43, 40.85)	0.178
Missing	0		0			
**Sport physical activity**	164		222			
No		155 (94.5)		197 (97.8)	REF	REF
Yes		9 (5.5)		5 (2.2)	2.57 (0.84, 7.84)	0.084
Missing	0		5			
**Walking behaviour more than 10 mints per day**	164		225			
No		66 (40.2)		114 (50.7)	REF	
Yes		98 (59.8)		111 (49.3)	1.52 (1.01, 2.29)	0.042
Missing	0		2			
**TV viewing time / per day**	145		206			
0–3 Hours		98 (67.6)		140 (68)	REF	
4+ Hours		47 (32.4)		66 (32)	1.01 (0.64, 1.60)	0.941
Missing	19		21			
**Sitting time / per day**	146		202			
0–4 Hours		61 (41.8)		100 (49.5)	REF	
5+ Hours		85 (58.2)		102 (50.5)	1.36 (0.88, 2.09)	0.154
Missing	18		25			
**Health seeking behaviours**						
**Hypertension**	164		227			
No		7 (4.3)		13 (5.7)	REF	
Yes		157 (95.7)		214 (94.3)	1.36 (0. 53-3.49)	0.518
Missing	0		0			
**Chronic disease diagnosis**	147		214			
No		147 (76.2)		159 (74.3)	REF	
Yes		35 (23.8)		55 (25.7)	0.90 (0.55, 1.47)	0.683
Missing	17		13			
**Self-reported health status**	164		226			
Very good or good		126 (76.8)		170 (75.2)	REF	
Fair or poor		38 (23.2)		56 (24.8)	0.91 (0.57, 1.46)	0.714
Missing	0		1			
**Self-reported health status compared with 12 months**	162		223			
Better or same		111 (68.5)		152 (68.2)	REF	
Worse		51 (31.5)		71 (31.8)	0.98 (0.63, 1.52)	0.941
Missing	2		4			
**Visited health services**	101		147			
Illness or injury		13 (12.9)		15 (10.2)	REF	
Other services		88 (87.1)		132 (89.8)	0.76 (0.34, 1.69)	0.514
Missing	63		80			

*REF* Reference category for statistical analysis, *CI* Confidence interval, *BMI* Body mass index

### Predictors of poorly controlled diabetes

#### Bivariate analyses

The key predictors identified from the bivariate analysis (Table 3) for the association with poor glycaemic control (*p* <  0.25) among type 2 diabetes, were marital status, educational level, dietary fat intake, fruit and vegetable intake, fast food intake, occupation and sport physical activity, walking behaviour and sitting periods.

### Multivariate analysis

The final multivariate model found that a dietary intake of three or more portions of fruit and vegetables was the only significant predictor in the final model associated with poorly controlled diabetes. All other predictor variables excluded earlier (*p* > 0.25) were included back in this model but none of them were retained. Intuitively, individuals who consume higher portions of fruit and vegetable should be expected to have better glycaemic control; however the results here show the opposite. One possibility to explain this is that the higher fruit intake was associated with other variables that can be more linked to poor glycaemic control (e.g. To investigate this we looked at the correlations between higher fruit and vegetable intake and overweight or obesity, animal fat or fast food consumption, no work, sport or walking physical activity and long TV viewing or sitting time). However, none of these correlations were found to be significant. Beta coefficient percentage change between the largest and smallest model variable from the model was less than 10% (provided in Additional file 4), which indicates lack of influence on other variables.

## Discussion

### Principal findings

Our study found that the prevalence of type 2 diabetes is higher among older individuals, particularly in those over 54 years and among males compared to females. Furthermore, being overweight, and having hypertension and chronic diseases such as asthma and heart failure are prevalent among patients with type 2 diabetes in Saudi Arabia. Patients with type 2 diabetes are more likely to report their ill-health or their health being worse compared to 1 year ago. On the other hand, the older individuals are more likely to have chronic diseases. When it came to physical activity, it was less likely for people, who walked in particular to have type 2 diabetes, but the same correlation could not be established with other indicators of better physical activities. The risk of having type 2 diabetes or poor glycaemic control was associated with low fast food and high fruit consumption, the opposite finding observed in studies elsewhere [30, 31].

### Predictors of type 2 diabetes

In this study, 35% of those aged 55 years and older had type 2 diabetes and 59% of those with type 2 diabetes were male. These findings agree with a study published in 2010 by the International Diabetes Federation (IDF) [32] suggesting that type 2 diabetes in Saudi Arabia (among other Middle Eastern countries) was expected to double by 2030 in association with the expected higher mean age. On the other hand, a study published in 2004, which included more than 17 thousand participants from Saudi Arabia above the age of 30 concluded that nearly 24% of Saudi’s had either type 1 or type 2 diabetes, with higher prevalence among males [33]. Some of the discrepancies between these studies and the findings here can be attributed to inclusion of type 1 diabetes in the “no type 2 diabetes group” in this study, while the distinction of type 1 and type 2 diabetes is not always made clear in other studies. However, the findings of this study on the higher prevalence of type 2 diabetes among the older age groups is consistent with global surveys. In a survey that included 111 countries, type 2 diabetes was concentrated among males between the age of 65 and 69, and 10 years later among females [34].

Being overweight or obese in particular, is thought to be the greatest risk factor for type 2 diabetes in Saudi Arabia [35, 36]. The final model of associations with type 2 diabetes supports this finding. Being overweight and obesity are known to be associated with other factors such as unhealthy diet and sedentary lifestyle. Our results found these lifestyle factors were significant at the bivariate level, but they were not retained in the final model. However, the final model showed that participants who reported walking for more than 10 min per day were less likely to report type 2 diabetes. This finding agrees with several reports that link walking behaviour with enhanced insulin sensitivity and glucose metabolism [37]. In a previous pilot study in Saudi Arabia on type 2 diabetes self-management that depended only on encouraging participants to walk more frequently, the participants had a significant improvement in their glycaemic control [38]. This suggests that the high percentage of people being overweight or obese has an important link to the high incidence of type 2 diabetes in adults living in Saudi Arabia, but also suggests walking to be more suited to the community there to counter both type 2 diabetes and weight gain, rather than other measures such as diet control or vigorous physical activities.

Frequent reports suggest that between 50 to 80% of diabetes patients have hypertension [39–41]. One of the significant associations found in the final model for type 2 diabetes was hypertension; participants with hypertension were three times more likely to report type 2 diabetes. This finding is in keeping with other communities outside Saudi Arabia [39, 40], nevertheless suggest that a focus should be given in any future type 2 diabetes self-management programme in Saudi Arabia, on the high risk of developing hypertension and how to minimise such risk. Guidelines for diabetes care recommend at least an annual check for blood pressure for those diagnosed with type 2 diabetes [41].

In addition to hypertension, the final model factors associated with having type 2 diabetes include chronic disease. The chronic disease category in this analysis included anyone with asthma, different chronic heart disease conditions, chronic renal disease, cerebral infarction or high cholesterol blood level. Diabetes is widely associated with neuropathy, chronic renal disease, adult blindness, fatty liver and chronic cardiovascular disease [42]. This association adds to the complicated nature of diabetes and is at the core of diabetes self-management [43]. This bidirectional relationship means that patients with type 2 diabetes should be educated on making health choices that can lower the risk of other chronic diseases and vice versa.

The type 2 diabetes final model showed that participants who report a reduction in their health status compared to 12 months ago were more likely to also report having type 2 diabetes. This can be partially attributed to the above mentioned association of diabetes with chronic illness [44]. But more importantly may reflect poor self-management of type 2 diabetes in Saudi Arabia.

A significant association between type 2 diabetes and lower frequency of fast food intake was found, which was unexpected. Evidence from previous studies suggests a higher risk of type 2 diabetes among those who consume fast food [45]. Potential explanations for this contradictory finding could be that this behaviour of less fast food intake was recently acquired after the patients had become aware of their diabetes [46].

This study shows the high prevalence of being overweight among patients with type 2 diabetes, but also the high prevalence of being overweight in the overall population, which correlates with the high prevalence of prediabetes among Saudi nationals, reported elsewhere [35, 47]. Although not conclusive in this study, healthy nutritional behaviours may not be acquired early enough and perhaps only after the diagnosis of diabetes. This suggests that programmes such as “let’s prevent diabetes” could be more suited for the local community and self-management of glucose level for individuals at risk of type 2 diabetes can be as important as self-management of the condition.

### Predictors of poor glycaemic control

Increased fruit and vegetable intake was the only variable to be significantly associated with poor glycaemic control following multivariate analysis. However, the finding that higher fruit and vegetable intake is associated with poorer glycaemic control is unexpected and contradicts previous research illustrating that a healthy diet is beneficial to health and reduces the risk of type 2 diabetes [48]. Potential explanations for this finding could be relevant to the higher frequency of diabetes symptoms among those with poor glycaemic control and that, similar to the justification provided above for lower fast food intake, higher fruit and vegetable intake behaviour may have been recently acquired but not necessary an overall healthier diet behaviour [46]. Nevertheless, fruits with high glycaemic index can be associated with poor glycaemic control [49]. SHIS did not question the types of fruits consumed by each participant, however it has been reported that the average individual consumption of dates in Saudi Arabia is around 122 g per day [50], which is equivalent to additional 338 kcal per day [51]. A significant association between glycaemic control and BMI and/or physical exercise was expected; however such relations were not evident in this study. However, only 41% of the participants with type 2 diabetes gave a blood sample. It may be fair to assume that those who attended the clinics are particularly interested in monitoring their health parameters compared to those who did not attend for the blood sample collection, which may have carried out an intrinsic bias in the data.

### Strengths and limitations

The strength of this study is that it was based on the SHIS which has included a large sample size from each of the 13 regions in Saudi Arabia. Obtaining the full data of the SHIS has supported running in depth analysis to address the aims of this study. However, the original SHIS survey was not designed as a needs-assessment study for diabetes self-management. The questions in the SHIS did not explore further aspects associated with a needs assessment such as accessibility to health care, availability of physical exercise facilities, and patients’ awareness of type 2 diabetes, its complications and management. The data was cross sectional and so causality cannot be assessed, and only half of the participants diagnosed with type 2 diabetes had blood samples taken at the clinic. In addition, some of the data was missing. Also, the survey was mainly based on self-reported assessments, which are known to have bias, when reporting undesirable lifestyle stigmas including unhealthy nutritional habits or lack of physical activity [52].

### Implications for future research

In accordance with Kumpfer’s cultural adaptation framework [19] the next stage if the programme of research is to, based on these findings, investigate comprehensive interventions in self-management programmes for type 2 diabetes in Saudi Arabia. Focus groups will be conducted to further explore the needs of older adults, weight management and managing comorbidities, such as hypertension and patient awareness of diabetes and its complications. Other sociodemographic and clinical factors not included in this study should be the subject of future studies, such as family history, income, disabilities, vitamin deficiencies, stress and depression.

## Conclusion

Our findings reflect specific priorities, including age, BMI and blood pressure, for the Saudi community that merit further investigation to fully understand the needs of the Saudi type 2 diabetes population and that should be taken into account in the development of a self-management programme for people with type 2 diabetes in Saudi Arabia. A focus should be made on the best approach to help older individuals make changes to their persistent habits and provide them with help to make sustainable lifestyle behaviour changes that are tailored to their age, but also to their likely comorbid chronic health conditions, especially hypertension [53]. Encouraging Saudis on walking, in particular, could be developed into a long-lasting and effective habit across a person’s lifetime for protection against type 2 diabetes. It is likely that Saudis would commit to better and healthier routines after being diagnosed with type 2 diabetes, but may ignore alarming signs prior to this.

## Supplementary information

**Additional file 1.**

**Additional file 2 **Type 2 diabetes analysis: Beta percentage change between largest and smallest models. Largest model refers to the first multivariate model with all variables included. Smallest model refers to the model with the five statistically significant (*p* <  0.05) variables.

**Additional file 3.** Results of Multivariate Analysis of Baseline Factors and Their Interactions.

**Additional file 4 **Glycaemic control analysis: Beta percentage change between largest and smallest models. Largest model refers to the first multivariate model with all variables included Smallest model refers to the model with one remaining statistically significant (*p* <  0.05) variable.

**Additional file 5.**

**Additional file 6.**

## Data Availability

The dataset for SHIS can be made available through direct request to MOH in Saudi Arabia. On line extract of SHIS are available in this publication and other publications that have used SHIS. Data analysis and further details of current study are available from the corresponding author upon reasonable request.
