# Supplementary material for: Correlates of type 2 diabetes and glycaemic control in adults in Saudi Arabia a secondary data analysis of the Saudi health interview survey
Source: BMC Public Health. 2020 Apr 17;20:515. doi: 10.1186/s12889-020-08597-6 (PMC7164173; doi:10.1186/s12889-020-08597-6)
Supplement: Supplementary file 1 — Additional file 1. [file 12889_2020_8597_MOESM1_ESM.docx]

| **Variables** | **New response options** | **Original variable response categories** | **Data manipulation and** **the new categories** |
| --- | --- | --- | --- |
| **Sociodemographic** | | | |
| Gender | Male =1  Female=2 | Male =1  Female=2 | No data manipulation required form original variable |
| Age  Educational Level | 15-54=1  ≥55 =2  Primary school or less=1  Elementary or high school or  college degree or higher education completed=2 | Age in years specified in open text field  Don’t know = 77  Declined to response = 88  Can’t read or write=1, Can read and write=2, Primary school completed=3, Intermediate school completed=4, High school completed=5 College/University completed=6 , Post graduate degree=7, Technical training=8 Don’t know=77 ,Decline to respond=88 | categories age continuous variable to one of two groups  Collapse education variables to:  1= Primary school or less  2= Elementary or high school or college / university or post graduate degree, and technical training in College degree or higher education completed |
| marital status | Married=1  Not married=2 | Never married=1, Currently married=2 Separated=3, Divorced=4 ,Widowed=5 Don’t know=77 , Decline to respond=88 | Combine marital variable in two:  1= Married  2= Not married |
| **History of diagnosis** | | | |
| height  weight | Normal weight=1  Overweight or obese=2 | Enter height (cm): Specify in open text field Declined measurement or could not measure participant’s height=88  Enter weight (kg): Specify in open text field Too heavy for scale=2 Declined measurement or could not measure participant’s height=88 | Use weight and height variables to calculate BMI  calculate BMI as weight (kg)/ height^2^(m^2^)  BMI classified into:  1= normal weight, if  BMI 18.5 to 24.9;  2= overweight, if  BMI 25.0 to 29.9;  AND obese, if BMI  greater than or equal to 30.0 |
| blood pressure | No=0  Yes=1 | Yes=1 No=0  Don’t know=77 Decline to respond=88 | No data manipulation required form original variable |
| Chronic Diseases  Stroke?  Mi?  CHF?  AFIB?  Asthma?  Renal Failure  Hypercholesterolemia? | No=0  Yes=1 | 1=Yes  0=No  77=Don’t know  88=Decline to respond | Yes to chronic disease if:  cerebral infection,  myocardial infarction,  heart failure, atrial fibrillation,  asthma, renal failure,  and hypercholesterolemia, = Yes  none of above conditions reported = No |
| **General health** | | | |
| Self-related health | Very good or good =1  Fair or poor =2 | 1=Excellent  2=Very good  3=Good  4=Fair  5=Poor  77=Don’t know  88=Decline to respond | collapse self-related health variable into two categories:  1= Very good or good  2= Fair or poor |
| Compared with 12 months ago | Better or same =1  Worse=2 | 3=Better  1=Worse  2=About the same  77=Don’t know  88=Decline to respond | No data manipulation required form original variable |
| **Lifestyle** | | | |
| Do you smoke | No=0  Yes=1 | Yes=1  No=0  Don’t know=77  Decline to respond=88 | If answer 0=No that mean never smoker, if answer 1= Yes, go to the smoking  status question if answer 0= No that mean previous smoker.  If answer 1= Yes that mean current smoker |
| Smoking current | No=0  Yes=1 | Yes=1  No=0  Don’t know=77  Decline to respond=88 |  |
| **Diet** | | | |
| Fat consumption | Vegetable or olive oils =1  Animal fat or margarine or none in particular =2 | Vegetable oil=1  Olive oil=2  Butter or ghee=3  Margarine=4  Other, please specify=5 If Other, specify in open text field  None in particular=6  None used=7  Don’t know=77  Decline to respond=88 | Collapse diet fat variable into two categories:  Vegetable or olive oils =1  Butter or ghee to Animal fat =2 |
| Fruit and vegetables intake | 0 – 2 =1  3+ =2 | Number of servings=1 Specify in open text field  Don't Know=77  Decline to respond=88 | Combine fruits and vegetables diet variable , categories diet fruit and vegetables serving continuous variable to one of two groups:  Daily serving fruits and vegetables and  will collected, if 0-2 times =1, if serving to  3 times or above per day= 2 |
| Red meat, pro meat, and chicken serving | 0-7 =1  8+ =2 | Number of days=1  Don't Know=77  Decline to respond=88 | Combine serving how many days per week eat of meat,  processed meats, and chicken  categories diet meat, processed meats, and chicken continuous variable to one of two groups:  will collected, if eat at lest 0-7 time per week=1,  if 8 times or above per week =2, |
| Eat fast food per week | 0-1 =1  2+ =2 | Number of meals=1  Don't Know=77  Decline to respond=88 | categories diet fast food continuous variable to one of two groups:  if eat at lest 1 day per week=1,  if 2 days or above per week =2, |
| **Sedentary** | | | |
| Hours spent watch tv | 0-3 =1  4+ =2 | Enter number of hours___________=1  Don’t know=77  Decline to respond=88 | categories tv comp time continuous variable to one of two groups:  if spent at least 0-3 hours per day=1,  if 4 hours or above per day =2, |
| Hours spent sitting | 0-4=1  5+ =2 | Hours per day=1  Minutes per day=2  Don’t know=77  Decline to respond=88 | categories sitting time continuous variable to one of two groups:  if spent at lest 0-4 hours per day=1,  if 5 hours or above per day =2, |
| **Physical activity** | | | |
| Physical mod activity sport | No=0  Yes=1 | Number of days=1  Don’t know=77  Decline to respond=88  Hours per day=1  Minutes per day=2  Don’t know=77  Decline to respond=88 | categories phy sport days and time continuous variable to  combined two variables how many days per week and how many time a day then counted as mints per week.  According WHO guideline use 150 mints for moderate and 75 mints for vigorous.=1 that mean Yes , if not achieved the minimum for those =0 that mean No |
| Physical vigactivity sport | No=0  Yes=1 | Number of days=1  Don’t know=77  Decline to respond=88  Hours per day=1  Minutes per day=2  Don’t know=77  Decline to respond=88 |  |
| Physical guide sport | No=0  Yes=1 |  | If moderate or vigorous physical activity Yes then physical activity guidelines for sport achieved, if No then physical activity guidelines for sport not achieved. |
| Physical mod activity work | No=0  Yes=1 | Number of days=1  Don’t know=77  Decline to respond=88  Hours per day=1  Minutes per day=2  Don’t know=77  Decline to respond=88 | categories phy sport days and time continuous variable to  combined two variables how many days per week and how many time a day then counted as mints per week.  According WHO guideline use 150 mints for moderate and 75 mints for vigorous.=1 that mean Yes , if not achieved the minimum for those =0 that mean No |
| Physical vigactivity work | No=0  Yes=1 | Number of days=1  Don’t know=77  Decline to respond=88  Hours per day=1  Minutes per day=2  Don’t know=77  Decline to respond=88 |  |
| Physical guide work | No=0  Yes=1 |  | If moderate or vigorous physical activity Yes then physical activity guidelines for work achieved, if No then physical activity guidelines for work not achieved |
| Walking behaviour more than 10 mints per day | No=0  Yes=1 | Yes=1  No=0  Don’t know=77 Decline to respond=88 | No data manipulation required form original variable |
| History of diagnosis with diabetes | | | |
| Diabetes | No=0  Yes=1 | 1=Yes  0=No  77=Don’t know 88=Decline to respond | No data manipulation required form original variable |
| Type of diabetes | Type 1=1  Type 2=2 | 1=Type 1  2=Type 2  77=I don’t know  88=decline to respond | No data manipulation required form original variable |
| Treatment of diabetes | Uncontrolled (Not treated) =1  Controlled (Treated)=2 | Yes=1  No=0  Don’t know=77  Decline to respond=88 | Prescribed treated uncontrolled or controlled, combined three variables, if prescribed treated insulin or drugs (medication) or special prescribed diet then treated controlled, if not prescribed any of them then untreated controlled |
| Measure HbA1c | ≤ 6.99 %=1  ≥7.0 % = 2 | Enter HgbA1C (%):  HgbA1C not measured=0 | categories HbA1c continuous variable to  defined the result to two, if between 6.5-6.99% diabetic with good glycaemic control=1  If ≥7.0% diabetic with poor glycaemic control=2 |
| Health seeking | | | |
| Use health services | Within the last 2 years for illness or injury=1  Within the last 2 years for other services=2 | 1=Enter year  995=I have never been to a hospital or clinic or doctor for medical attention.  77=Don’tknow  88=Decline to respond  For reson  Illness=1  Injury=2  Immunization=3  Other preventive service=4  Other, please specify=5 If Other, specify in open text field  Don’t know=77  Decline to respond=88 | Combined two variables last time of visiting and respond if visit clinic or hospital or other health professional |
