# Supplementary material for: Correlates of type 2 diabetes and glycaemic control in adults in Saudi Arabia a secondary data analysis of the Saudi health interview survey
Source: BMC Public Health. 2020 Apr 17;20:515. doi: 10.1186/s12889-020-08597-6 (PMC7164173; doi:10.1186/s12889-020-08597-6)
Supplement: Supplementary file 3 — Additional file 3. Results of Multivariate Analysis of Baseline Factors and Their Interactions. [file 12889_2020_8597_MOESM3_ESM.docx]

| **Variables** | **p-value** |
| --- | --- |
| Gender | < 0.001 |
| Age | < 0.001 |
| BMI | < 0.001 |
| Hypertension | < 0.001 |
| Chronic disease diagnosis | < 0.001 |
| Self-reported health condition compared with 12 months | < 0.001 |
| Dietary fast food intake | < 0.001 |
| Walking behaviour | < 0.001 |
| Gender (male)*Age (older) | 0.532 |
| Gender (male)*BMI (obese) | 0.060 |
| Gender (male)*Hypertension (yes) | 0.185 |
| Gender (male)* Chronic disease diagnosis (yes) | 0.915 |
| Gender (male)* Self-reported health condition compared with 12 months (worse) | 0.422 |
| Gender (male)* Dietary fast food intake (no) | 0.262 |
| Gender (male)* Walking behaviour (yes) | 0.670 |
| Age (older)*BMI (obese) | 0.596 |
| Age (older)* Hypertension (yes) | 0.267 |
| Age (older)* Chronic disease diagnosis (yes) | < 0.001 |
| Age (older)* Self-reported health condition compared with 12 months (worse) | 0.002 |
|  |  |
|  |  |
| Age (older)* Dietary fast food intake (no) | 0.575 |
| Age (older)* Walking behaviour (yes) | 0.861 |
| BMI (obese)* Hypertension (yes) | 0.449 |
| BMI (obese)* Chronic disease diagnosis (yes) | 0.847 |
| BMI (obese)* Self-reported health condition compared with 12 months (worse) | 0.592 |
| BMI (obese)* Dietary fast food intake (no) | 0.472 |
| BMI (obese)* Walking behaviour (yes) | 0.857 |
| Hypertension (yes)* Chronic disease diagnosis (yes) | 0.743 |
| Hypertension (yes) * Self-reported health condition compared with 12 months (worse) | 0.956 |
| Hypertension (yes)* Dietary fast food intake (no) | 0.504 |
| Hypertension (yes)* Walking behaviour (yes) | 0.097 |
| Chronic disease diagnosis (yes)* Self-reported health condition compared with 12 months (worse) | 0.215 |
| Chronic disease diagnosis (yes) * Dietary fast food intake (no) | 0.296 |
| Chronic disease diagnosis (yes)* Walking behaviour (yes) | 0.632 |
| Self-reported health condition compared with 12 months (worse)* Dietary fast food intake (no) | 0.061 |
| Self-reported health condition compared with 12 months (worse)* Walking behaviour (yes) | 0.441 |
| Dietary fast food intake (no) * Walking behaviour (yes) | 0.745 |
