# Supplementary material for: Correlates of type 2 diabetes and glycaemic control in adults in Saudi Arabia a secondary data analysis of the Saudi health interview survey
Source: BMC Public Health. 2020 Apr 17;20:515. doi: 10.1186/s12889-020-08597-6 (PMC7164173; doi:10.1186/s12889-020-08597-6)
Supplement: Supplementary file 4 — Additional file 4 Glycaemic control analysis: Beta percentage change between largest and smallest models. Largest model refers to the first multivariate model with all variables included Smallest model refers to the model with one remaining statistically significant (p < 0.05) variable. [file 12889_2020_8597_MOESM4_ESM.docx]

| **Variable** | **Beta; largest model** | **Beta; smallest model** | **% change between models** |
| --- | --- | --- | --- |
| Dietary fruits and vegetables intake | -0.749 | -0.684 | 9.502924 |
