# Supplementary material for: Correlates of type 2 diabetes and glycaemic control in adults in Saudi Arabia a secondary data analysis of the Saudi health interview survey
Source: BMC Public Health. 2020 Apr 17;20:515. doi: 10.1186/s12889-020-08597-6 (PMC7164173; doi:10.1186/s12889-020-08597-6)
Supplement: Supplementary file 5 [file 12889_2020_8597_MOESM5_ESM.docx]

Kingdom of Saudi Arabia
Ministry of Health
King Fahad Medical City
(162)

وزارة الصحة
مدينة الملك فهد الطبية
King Fahad Medical City

المملكة العربية السعودية
وزارة الصحة
مدينة الملك فهد الطبية
(١٦٢)

IRB Registration Number with KACST, KSA:	H-01-R-012
IRB Registration Number with OHRP/NIH, USA:	IRB00010471
Approval Number Federal Wide Assurance NIH, USA:	FWA00018774

February 27, 2017
IRB Log Number: 17-063E
Department: External
Category of Approval: EXEMPT

Dear Thamer Alslamah,

I am pleased to inform you that your submission dated February 19, 2017 for the study titled '**Cultural Adaptation in the Self-Management of Type 2 Diabetes in Saudi Arabia**' was reviewed and was approved. Please note that this approval is from the research ethics perspective only. You will still need to get permission from the head of department or unit in KFMC or an external institution to commence data collection.

We wish you well as you proceed with the study and request you to keep the IRB informed of the progress on a regular basis, using the IRB log number shown above.

Please be advised that regulations require that you submit a progress report on your research every 6 months. You are also required to submit any manuscript resulting from this research for approval by IRB before submission to journals for publication.

As a researcher you are required to have current and valid certification on protection human research subjects that can be obtained by taking a short online course at the US NIH site or the Saudi NCBE site followed by a multiple choice test. Please submit your current and valid certificate for our records. Failure to submit this certificate shall a reason for suspension of your research project.

If you have any further questions feel free to contact me.

Sincerely yours,

Prof. Omar H. Kasule
Chairman, Institutional Review Board (IRB)
King Fahad Medical City, Riyadh, KSA
Tel: + 966 1 288 9999 Ext. 26913
E-mail: okasule@kfmc.med.sa

K F M C
Approved
27 FEB 2017
I R B

وزارة الصحة
Ministry of Health

To whom it may concern

Candidate name: Thamer Alslamah
Project Title: Need Assessment of Cultural Adaptation of Self-Management of Type 2 Diabetes in Saudi Arabia
Ethical approval number: FWA 00018774

This is to confirm that the National Diabetes Prevention and Control Program at Ministry of Health has reviewed the above-mentioned paper and has agreed on proceeding submission for publication in scientific journal.

Director of National Diabetes Prevention and Control Program

Dr. Fatima Al Slail

المملكة العربية السعودية
وزارة الصحة
وكالة الوزارة للصحة العامة
الإدارة العامة لمكافحة الأمراض الوراثية والمزمنة
