# Supplementary material for: Correlates of type 2 diabetes and glycaemic control in adults in Saudi Arabia a secondary data analysis of the Saudi health interview survey
Source: BMC Public Health. 2020 Apr 17;20:515. doi: 10.1186/s12889-020-08597-6 (PMC7164173; doi:10.1186/s12889-020-08597-6)
Supplement: Supplementary file 6 [file 12889_2020_8597_MOESM6_ESM.docx]

Dear Dr Craig Melville
MVLS College Ethics Committee

Project Title: *Cultural Adaptation in the Self-Management of Type 2 Diabetes in Saudi Arabia*
Project No: *200160103*

The College Ethics Committee has reviewed your application and has agreed that there is no objection on ethical grounds to the proposed study. We are happy therefore to approve the project, subject to the following conditions:

- Project end date: as per application.

- The data should be held securely for a period of ten years after the completion of the research project, or for longer if specified by the research funder or sponsor, in accordance with the University's Code of Good Practice in Research: (http://www.gla.ac.uk/media/media_227599_en.pdf)

- The research should be carried out only on the sites, and/or with the groups defined in the application.

- Any proposed changes in the protocol should be submitted for reassessment, except when it is necessary to change the protocol to eliminate hazard to the subjects or where the change involves only the administrative aspects of the project. The Ethics Committee should be informed of any such changes.

- You should submit a short end of study report to the Ethics Committee within 3 months of completion.

Yours sincerely

Dr Terry Quinn

Terry Quinn
MD, FRCP, BSc (hons), MBChB (hons)
Senior Lecturer / Honorary Consultant

College of Medicine, Veterinary & Life Sciences
Institute of Cardiovascular and Medical Sciences
New Lister Building, Glasgow Royal Infirmary
Glasgow
G31 2ER
terry.quinn@glasgow.gla.ac.uk
Tel – 0141 201 8519

The University of Glasgow, charity number SC004401
